# The Effects of FKBP12 Single-Dose Plasmid Oral Preparation on the Alkaline Phosphatase Activity, Mortality Rate, and Cocoon Quality of Bivoltine Jinqiu × Churi Silkworms

**DOI:** 10.3390/ijms27010237

**Published:** 2025-12-25

**Authors:** Jiaqi Chen, Ziyu Song, Wenbin Li, Haoyang Feng, Yani Wang, Dajin Wang, Si Li

**Affiliations:** College of Life Sciences and Medicine, Zhejiang Sci-Tech University, Hangzhou 310018, China; 2022332864003@mails.zstu.edu.cn (J.C.); 2022332864061@mails.zstu.edu.cn (Z.S.); 2023332864062@mails.zstu.edu.cn (W.L.); 2023332864067@mails.zstu.edu.cn (H.F.); 2023331200065@mails.zstu.edu.cn (Y.W.); wangdj@zstu.edu.cn (D.W.)

**Keywords:** silkworm, alkaline phosphatase, FKBP12, cocoon shell ratio, plasmid, oral delivery

## Abstract

Although naturally colored silk is an excellent textile material and biomedical material, some silkworm varieties—such as the bivoltine strain Jinqiu × Churi—exhibit a relatively low cocoon shell ratio, which limits their economic value. This study, therefore, aimed to develop a novel approach to enhance larval survival and cocoon quality by orally administering a single dose of a recombinant pIEx-1-*FKBP12B* plasmid to silkworm larvae at different instars. The optimal feeding larval stages and dosage were evaluated based on the enhancement of alkaline phosphatase activity, mortality rate, cocoon shell weight, cocoon shell ratio, and silk weight. The best results were observed in the group that received a single oral dose of 5 μg of recombinant pIEx-1-*FKBP12B* plasmid during the second instar. This group exhibited significantly higher alkaline phosphatase activity and fifth instar larval body length compared to the group receiving wild plasmid pIEx-1. The cocoon shell weight was 21.96% higher, the cocoon shell ratio was 2.5% higher, and the silk weight was 33.60% higher than that of the control group. The mortality rate was the lowest among all the groups, 36.89% lower than the control group. These findings suggest that oral administration of the pIEx-1-*FKBP12B* plasmid has significant potential for application in improving silk quality.

## 1. Introduction

Silk has found applications in the textile industry, biological medicine, and tissue engineering due to its extraordinary mechanical properties, biocompatibility, and biodegradability [1,2,3,4]. Naturally colored domestic silks (NCSs) are particularly prized for these applications as they eliminate the need for chemical dyeing [5]. These silks are produced by specific genes that transfer pigments from mulberry leaves to the silk gland, and exhibit a fundamental structure and properties similar to common white silk [6]. Jinqiu × Churi is a new silkworm variety developed at Zhejiang University, which received crop variety approval from Zhejiang Province in January 2017 (approval number: Zhejiang province 2017001). The cocoon of this variety is naturally yellow and is very popular because it does not require chemical dyeing [7]; however, it suffers from a relatively low cocoon shell ratio. The economic value of silkworm rearing is reflected in the proportion of cocoon shell to cocoon weight and the quality of the cocoon shell [8]. Previous experiments conducted by this research group demonstrated that upregulating the FK506 binding protein 12 (*FKBP12B*) gene (GenBank accession no. DQ443423) in both BmN cells and silkworm larvae significantly and rapidly enhances alkaline phosphatase (ALP) expression and activity. We also compared the effects of injecting and feeding silkworms with *FKBP12* plasmid reagents, finding that feeding was more effective, gentle, safe, and convenient.

ALP activity can serve as a biochemical index for the health and economic value of the economic insect *Bombyx mori* [9]. ALP is a key enzyme in silkworms, involved in the transfer and hydrolysis of phosphate groups, the detoxification of exogenous compounds, and resistance to pathogenic microorganisms [10,11]. By enhancing stress resistance, ALP activation contributes to both the survival and the silk production capacity of silkworms [12,13]. The silkworm possesses two forms of ALP: membrane-bound ALP (m-ALP), associated with digestion and nutrient absorption, and soluble ALP (s-ALP), which likely functions in ion balance [8,14]. Critically, ALP activity is closely and positively correlated with major economic traits, including cocoon shell weight and silk length, making it a reliable biochemical indicator for silkworm health and economic value [15]. Common approaches to enhance ALP, such as hormone administration, often only induce transient effects [16]. Therefore, more sustainable strategies to modulate this important enzyme are needed.

We focused on the FK506 binding protein 12 (FKBP12), which is an intracellular receptor for the immunosuppressant tacrolimus (FK506) and a 12 kDa protein widely involved in cellular processes [17]. FKBP12 possesses peptidyl-prolyl cis-trans isomerase (PPIase) activity, which plays a crucial role in regulating protein folding and phosphorylation [18]. In *Bombyx mori*, the FKBP12B isoform exhibits a distinct subcellular localization, being present in the nuclear membrane and nucleus, unlike its typical cytoplasmic localization in other species [19]. Furthermore, FKBP12B is highly expressed in the silk gland and intestine [20], suggesting a specific role in these tissues relevant to nutrient metabolism, gene regulation, and silk protein synthesis.

In mammals, FKBP12 serves as an inhibitor for bone morphogenetic protein (BMP) type I receptors (BMPRI). By using the FKBP12 inhibitor FK506 (tacrolimus) or knocking down FKBP12 with siRNA in mouse myoblasts, BMP signaling pathway activation can be induced, leading to a significant increase in ALP activity [21]. These experimental phenomena might be related to FKBP12’s regulation of calcium ion (Ca^2+^) concentration. The FKBP12 protein binds to calcineurin (CaN), thereby inhibiting the inositol 1,4,5-trisphosphate receptor (IP3R) to regulate calcium ion release [22]. FKBP12 also binds to the ryanodine receptor RyR1. Together, four RyR subunits and four FKBP12 subunits form a tetrameric complex [23], which plays a stabilizing role in the opening and closing of the Ca^2+^ release channel [24]. Ryanodine exhibits biphasic regulation: at nanomolar (nM) concentrations in mammalian muscle, it opens or locks the RyR/Ca^2+^ release channel, while at micromolar (μM) concentrations, it closes the channel [25]. The inositol 1,4,5-trisphosphate receptor (IP3R) and the ryanodine receptor (RyR) are the primary calcium channels on the endoplasmic reticulum, which are both regulated by FKBP12. Furthermore, changes in Ca^2+^ concentration are also closely associated with ALP activity [26,27]. There is a close regulatory relationship between calcium ions and ALP, which is mainly reflected in the following aspects: Ca^2+^ can directly enhance ALP activity, an effect that is particularly significant in various microorganisms. For example, in the study by Pandey et al., a systematic analysis of the response of diazotrophic cyanobacteria (including *Hapalosiphon intricatus* and *Calothrix brevissima*) to calcium ions showed that ALP activity increases with rising calcium ion concentration. This activation effect intensifies as the Ca^2+^ concentration increases, indicating that Ca^2+^ may directly bind to the active site of the enzyme, stabilize its three-dimensional conformation, or improve substrate accessibility, thereby optimizing catalytic efficiency. Further mechanistic studies reveal that calcium ions specifically bind to the EF-hand domain of ALP, inducing conformational changes and promoting the hydrolysis of phosphoester bonds [26]. In *Anabaena variabilis* [26] and fungi [27], the activity of calcium-dependent alkaline phosphatase is positively correlated with intracellular calcium levels. This may indirectly influence enzyme expression through the calmodulin/calcineurin signaling pathway, reduce the activation energy of the reaction, and enhance the conversion rate. This phenomenon is particularly prominent under phosphate-deficient conditions [26,27].

This study attempts to upregulate alkaline phosphatase activity by orally administering a recombinant plasmid of the *FKBP12B* gene to silkworm larvae in a single dose, aiming to enhance the survival rate of silkworms and improve the quality of cocoons and silk, while also investigating and recommending suitable larval stages and dosages.

## 2. Results

### 2.1. Analysis of q-PCR Results in Silkworm Larvae of Different Instars

**Figure 1:**
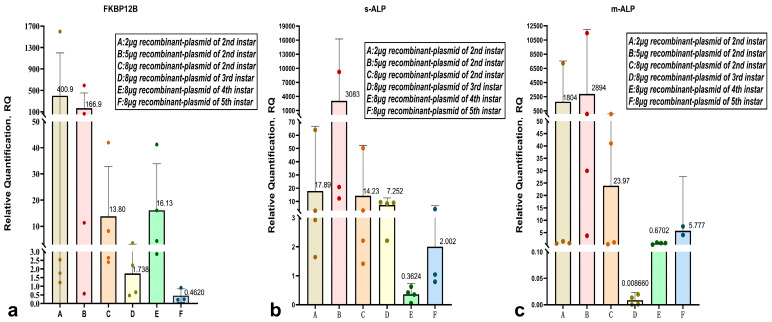
Relative expression analysis of *FKBP12* (GenBank accession no. DQ443423) (**a**) *s-ALP* (GenBank accession no. AB013386.1) (**b**) and *m-ALP* (GenBank accession no. D90454.4) (**c**) after 144 h plasmid feeding in different groups. The data are presented as the mean values ± SD, n = 4, in most groups. (Specifically, n = 3 in 5 μg RP of *s-ALP* and 8 μg RP of each gene). An RQ value greater than 1 indicates an upregulation of gene expression, and an RQ value less than 1 indicates a downregulation of gene expression.

Silkworms fed with pIEx-1-*FKBP12B* plasmid reagent at different doses during the 2nd instar were sampled on the 6th day. The average 2^−ΔΔCT^ value of *FKBP12B* at different doses (2 μg, 5 μg, 8 μg per silkworm) was 400.9, 166.9, and 13.8, respectively. Except for one sampled silkworm in the 5 μg group with a 2^−ΔΔCT^ value of 0.58, all other sampled silkworms had values greater than 1, indicating that the *FKBP12B* gene was upregulated in almost all sampled silkworms, with an upregulation rate of 93.75%. The relative expression level of the *FKBP12* gene was highest in the 2 μg recombinant plasmid group among all 2nd instar groups (Figure 1a).

**Figure 2:**
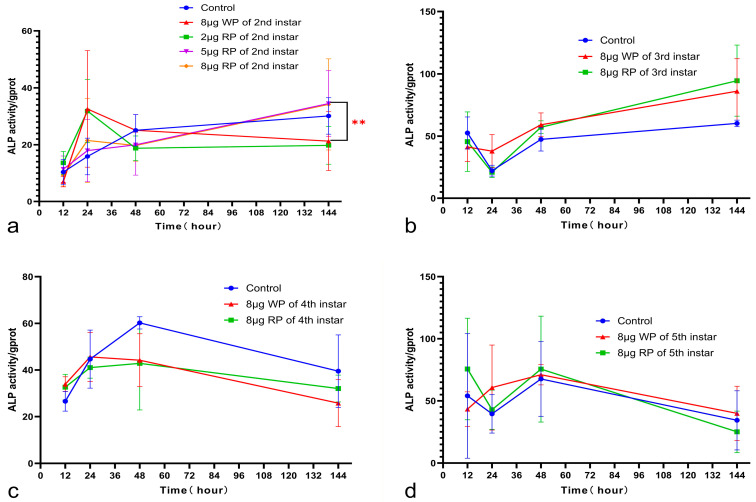
Analysis of alkaline phosphatase (ALP) activity in the feeding groups of the 2nd instar (**a**), 3rd instar (**b**), 4th instar (**c**), and 5th instar silkworm larvae (**d**) after 12 h, 24 h, 48 h, and 6 days of plasmid feeding. WP: wild plasmid, RP: recombinant plasmid. Data are presented as the mean values ± SD, n = 4 in each group. ** *p* < 0.01.

The relative expression levels of soluble alkaline phosphatase (*s-ALP****)*** and membrane-bound alkaline phosphatase (*m-ALP*) were significantly increased in all drug groups, with the highest levels observed in the 5 μg recombinant plasmid group (3083 and 2894) (Figure 1b,c). This result was consistent with the trend of alkaline phosphatase activity measured on the 6th day after feeding in the second instar (Figure 2).

The relative expression levels of the *FKBP12B* gene in the 8 μg drug groups fed during the second, third, and fourth instars were all greater than 1 (13.8, 1.738, and 16.13, respectively), while the relative expression level in the fifth instar feeding group was 0.462, indicating no increase. The relative expression levels of *s-ALP* and *m-ALP* in the 8 μg drug groups fed during the second to fifth instars were as follows: second instar feeding: 14.23 and 23.97; third instar feeding: 7.252 and 0.00866; fourth instar feeding: 0.3624 and 0.6702; fifth instar feeding: 2.002 and 5.777. The total relative expression levels of *s-ALP* and *m-ALP* were highest in the second instar feeding group, followed by the third instar feeding group. Feeding recombinant plasmid during the fourth instar did not increase the relative expression levels of *s-ALP* and *m-ALP* (Figure 1b,c).

### 2.2. Analysis of Alkaline Phosphatase Activity

The alkaline phosphatase activity change curve in the second instar feeding group showed that the control group and the wild plasmid control group had their own patterns of alkaline phosphatase activity changes, while the recombinant plasmid group had a different trend. The control group showed a gradual increase in alkaline phosphatase activity between 12 h and 144 h after feeding, with the rate of increase slowing down between 48 h and 144 h. The empty plasmid control group had the lowest alkaline phosphatase activity (6.991 ± 0.9231 gprot) at 12 h after feeding, peaked at 24 h (32.58 ± 11.83 gprot), and then gradually decreased between 24 h and 144 h, reaching 21.26 gprot at 144 h. The three recombinant plasmid groups showed an increase in alkaline phosphatase activity between 12 h and 24 h after feeding. Between 24 h and 48 h, except for the 5 μg recombinant plasmid group—which showed a slight increase—the other two drug groups showed a decrease. At 48 h after feeding, the alkaline phosphatase activities of the three recombinant plasmid groups tended to be similar, ranging from 18.74 to 19.97 gprot. Between 48 h and 144 h, all three drug groups showed an increasing trend. At 144 h after feeding, the alkaline phosphatase activities of two of the recombinant plasmid groups were higher than those of the control group and the empty plasmid group. The 5 μg recombinant plasmid group showed a highly significant difference in ALP activity compared to the wild plasmid group. The 8 μg recombinant plasmid groups fed during the second and third instars had higher alkaline phosphatase activities at 144 h after feeding than the control group and the empty plasmid group, while the 8 μg recombinant plasmid groups fed during the fourth and fifth instars had lower alkaline phosphatase activities at 144 h after feeding than the control group. From the perspective of alkaline phosphatase activity, feeding a *FKBP12B* plasmid drug during the early young stage of silkworms yielded better results (Figure 2).

### 2.3. Statistical Analysis of Larval Mortality Before Cocooning and Cocooning Success Rate

**Figure 3:**
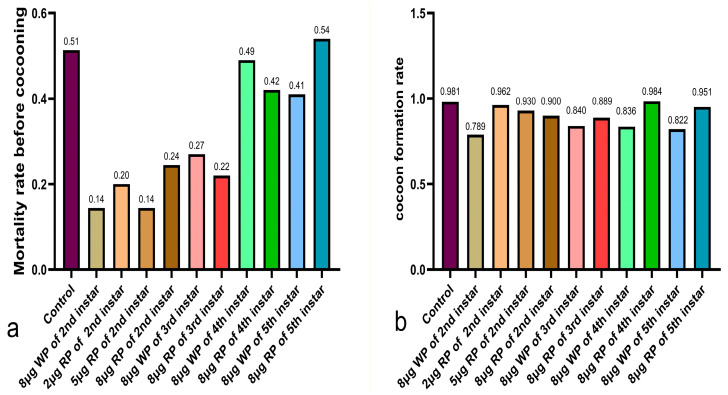
Analysis of mortality rate (**a**) and cocoon formation rate (**b**) of the feeding groups of the 2nd instar, 3rd instar, 4th instar, and 5th instar silkworm larvae after plasmid treatment. WP: wild plasmid, RP: recombinant plasmid. The larval mortality rate is the proportion of the number of all dead individuals during the larval stage in a given group to the initial number of larvae in the experiment. The cocoon formation rate is the ratio of successfully cocooned silkworms to surviving late fifth instar larvae in a specific group.

Except for the fifth instar feeding group with 8 μg recombinant plasmid, which had a high mortality rate of 54%, higher than the control group’s 51%, the mortality rates of the other second to fourth instar feeding groups were all lower than the control group. The mortality rates of the third and fourth instar 8 μg feeding groups fed with recombinant plasmid were lower than those fed with wild plasmid, with the third instar recombinant plasmid group had a mortality rate of 22%, 5% lower than the blank plasmid group, and the fourth instar recombinant plasmid group had a mortality rate of 42%, 7% lower than the blank plasmid group. The second instar feeding group performed the best overall, with mortality rates ranging from 14% to 24%, with the 8 μg wild plasmid feeding group and the 5 μg recombinant plasmid feeding group performing the best (Figure 3a).

After the larval stage, the cocooning success rate was highest in the blank control group at 98%. The cocooning success rates of the second to fifth instar feeding groups fed with recombinant plasmid were all higher than those fed with wild plasmid. The cocooning success rates of the second instar feeding groups fed with recombinant plasmid ranged from 90% to 96%, all higher than the 79% of the wild plasmid feeding group. The cocooning success rates of the third, fourth, and fifth instar feeding groups fed with recombinant plasmid were also higher than those of the same instar groups fed with wild plasmid (Figure 3b).

### 2.4. Statistical Analysis of the Fifth Instar Larval Body Length, Cocoon Length and Width, Cocoon Shell Weight, Pupa Weight, and Cocoon Shell Ratio

**Figure 4:**
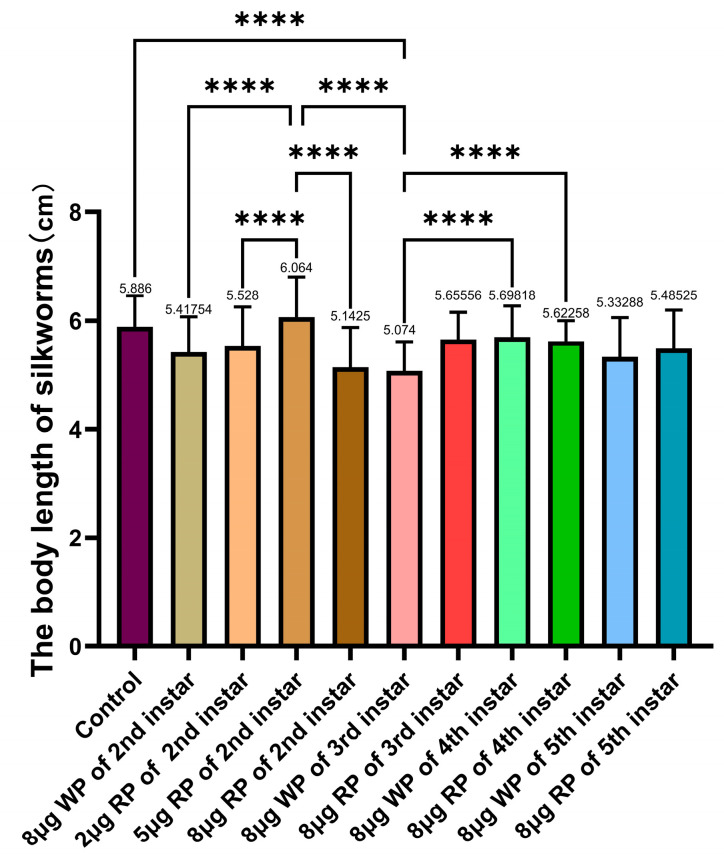
Body length of the 5th instar silkworm larvae following the plasmid feeding of the 2nd instar, 3rd instar, 4th instar, and 5th instar silkworm larvae. WP: wild plasmid, RP: recombinant plasmid. The body length of fifth-instar larvae was measured on day 5 of the fifth instar. Data are presented as the mean values ± SD, n = 49–72 of groups (all surviving individuals measured). **** *p* < 0.0001.

Among all the second instar feeding groups fed with recombinant plasmid, only the 5 μg feeding group had a fifth instar larval body length (6.06 cm) longer than the control group (5.89 cm). The control group even had a significantly longer larval body length than the 8 μg wild plasmid group (5.42 cm) and the 8 μg recombinant plasmid group (5.14 cm). The larval body length of the 5 μg recombinant plasmid group was the longest, significantly longer than the wild plasmid group, the 2 μg recombinant plasmid group, and the 8 μg recombinant plasmid group (5.53 cm and 5.14 cm). In the comparison of fifth instar larval body lengths among the second to fifth instar 8 μg feeding groups, all second to fifth instar 8 μg feeding groups fed with recombinant plasmid had shorter body lengths than the completely blank control group’s 5.89 cm. The third instar feeding group with 8 μg recombinant plasmid had a larval body length (5.66 cm) longer than the third instar feeding group with 8 μg wild plasmid (5.07 cm) and the second instar feeding group with 8 μg recombinant plasmid (5.14 cm). Feeding recombinant plasmid during the second and third instars of silkworm larvae resulted in longer fifth instar larval body lengths compared to feeding with blank plasmid. The best results were observed in the second instar feeding group with 5 μg recombinant plasmid, which had the longest larval body length of all the groups (Figure 4).

**Figure 5:**
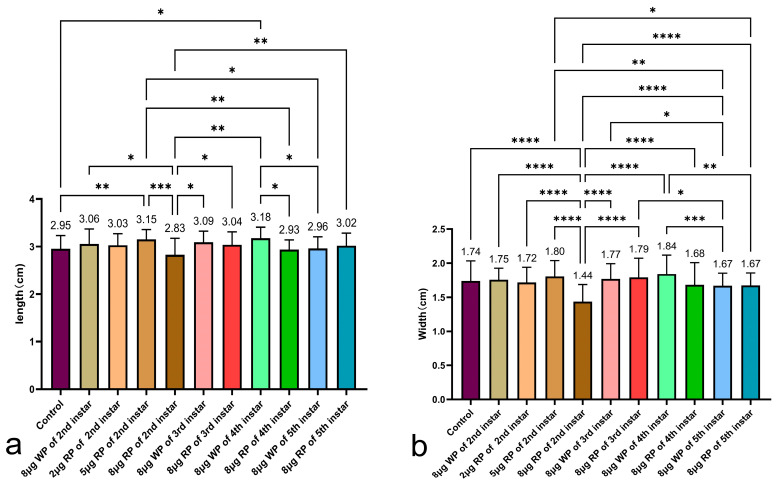
Analysis of cocoon shell length (**a**) and width (**b**). WP: wild plasmid, RP: recombinant plasmid. Data are presented as the mean values ± SD, n = 29–51 of groups (measuring all individuals that have successfully formed cocoons, excluding those used for silk reeling measurement). * *p* < 0.05, ** *p* < 0.01, *** *p* < 0.001, **** *p* < 0.0001.

In the measurement of cocoon shell length, except for the 8 μg feeding group, all other second instar feeding groups had longer cocoon shell lengths than the control group. The best results were observed in the second instar feeding group with 5 μg recombinant plasmid, which had the longest cocoon shell length (3.15 cm), significantly longer than the completely blank control group, the 8 μg recombinant plasmid drug group (2.95 cm, 2.83 cm). In the comparison of cocoon shell lengths among the second to fifth instar 8 μg feeding groups, the third and fifth instar feeding groups with 8 μg recombinant plasmid (3.04 cm and 3.02 cm, respectively) had slightly longer cocoon lengths than the control group (2.95 cm). The second and fourth instar feeding groups with recombinant plasmid had slightly shorter cocoon lengths than the control group (2.83 cm and 2.93 cm, respectively), and significantly shorter lengths than the same instar groups fed with the same amount of wild plasmid (Figure 5a).

In the measurement of cocoon shell width, only the 8 μg feeding group in the second instar had a cocoon shell width (1.44 cm) significantly shorter than the completely blank control group (1.74 cm), the blank plasmid control group, and the 2 μg and 5 μg recombinant plasmid drug groups (1.72 cm, 1.80 cm). The fourth instar feeding group with recombinant plasmid had a shorter cocoon width than the same instar group fed with the same amount of blank plasmid (1.68 cm and 1.84 cm, respectively) (Figure 5b).

**Figure 6:**
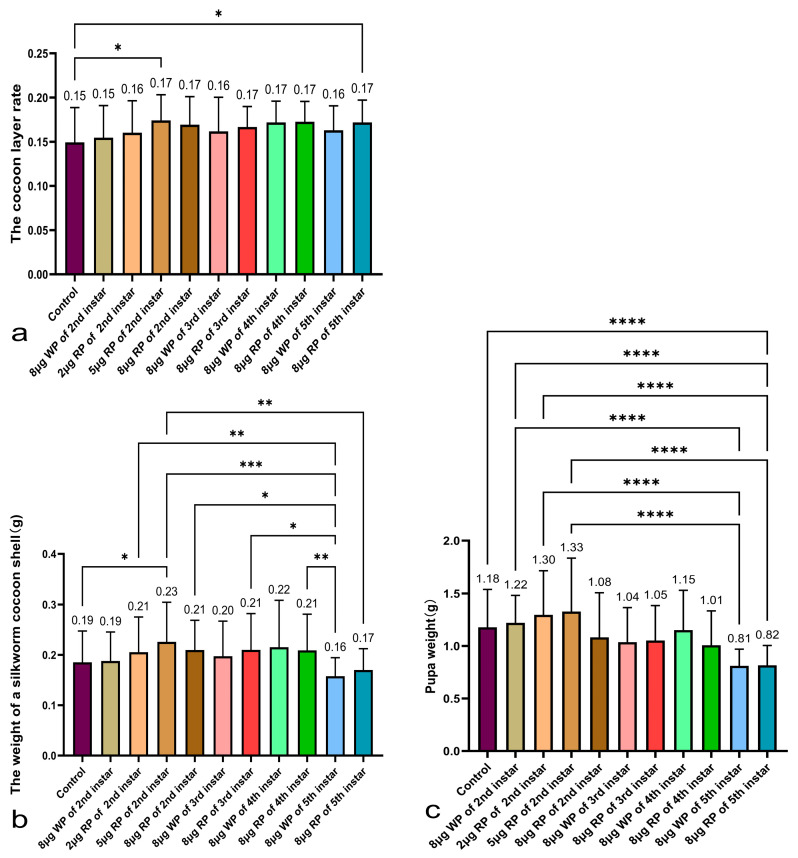
Analysis of the cocoon layer rate (**a**), cocoon shell weight (**b**), and weight of silkworm pupae (**c**). WP: wild plasmid, RP: recombinant plasmid. Data are presented as the mean values ± SD, n = 30–61 of groups (measuring all individuals that have successfully formed cocoons, excluding those used for silk reeling measurement). * *p* < 0.05, ** *p* < 0.01, *** *p* < 0.001, **** *p* < 0.0001.

All second instar feeding groups fed with recombinant plasmid had higher cocoon shell weights than the control group (0.186 g), with the second instar feeding group with 5 μg recombinant plasmid having the highest cocoon shell weight (0.226 g), higher than the wild plasmid group (0.188 g) and significantly higher than the control group. In the comparison of cocoon shell weights among the second to fifth instar 8 μg feeding groups, the second, third, and fourth instar feeding groups with 8 μg recombinant plasmid had higher cocoon shell weights (0.21 g, 0.21 g, 0.209 g) than the control group (0.185 g). The second and third instar feeding groups with 8 μg wild plasmid had lower cocoon shell weights (0.188 g, 0.197 g) than those of the recombinant plasmid group, and the fourth instar feeding group with 8 μg wild plasmid had higher cocoon shell weights (0.215 g) than the recombinant plasmid group (0.209 g). The fifth instar feeding group with recombinant plasmid had a lower cocoon shell weight (0.17 g), and the wild plasmid group had a higher cocoon shell weight (0.158 g) than the other instar groups and the control group (Figure 6b).

The second instar feeding groups with 2 μg and 5 μg recombinant plasmid, and 8 μg blank plasmid had higher pupa weights (1.30 g, 1.33 g, and 1.22 g, respectively) than the control group (1.177 g), while the 8 μg recombinant plasmid feeding group had a lower average pupa weight (1.08 g) than the completely blank control group. In the comparison of pupa weights among the second to fifth instar 8 μg feeding groups, all 8 μg feeding groups fed with recombinant plasmid had lower pupa weights (1.08 g, 1.05 g, 1.01 g, 0.82 g, respectively) than the control group (0.177 g). The fifth instar feeding group with recombinant plasmid had a pupa weight of 0.82 g, and the wild plasmid group had a pupa weight of 0.81 g, which are both significantly lower than the control group (Figure 6c).

All second instar feeding groups fed with recombinant plasmid had higher cocoon shell ratios than the control group (14.9%), with the second instar feeding group with 5 μg recombinant plasmid having the highest cocoon shell ratio (17.4%) among all the groups. In the comparison of cocoon shell ratios among the second to fifth instar 8 μg feeding groups, all 8 μg feeding groups fed with recombinant plasmid had higher cocoon shell ratios (second to fifth instars: 16.9%, 16.7%, 17.3%, and 17.2%, respectively) than the same instar groups fed with the same amount of wild plasmid (second to fifth instars: 15.5%, 16.2%, 17.2%, and 16.3%, respectively), and higher than the control group (14.9%) (Figure 6a).

### 2.5. Determination of Moisture Content and Re-Humidification Rate

**Figure 7:**
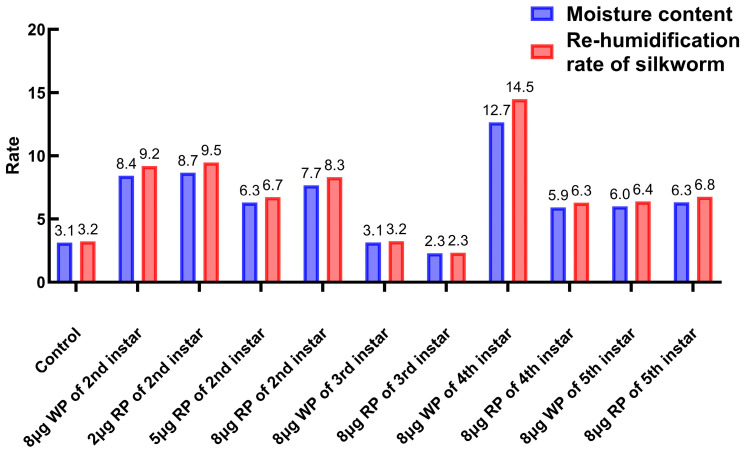
Determination of the moisture content and re-humidification rate of silkworm cocoons. WP: wild plasmid, RP: recombinant plasmid.

The moisture content of the control group was 3.12%. The moisture content of cocoons in the second instar feeding group with wild plasmid was 8.42%, while the moisture content of the second instar feeding groups with 2 μg, 5 μg, and 8 μg oral recombinant plasmid was 8.66%, 6.30%, and 7.67%, respectively. The moisture content of cocoons in the third instar feeding group with blank plasmid was 3.14%, while the oral recombinant plasmid group had a moisture content of 2.29%. The moisture content of cocoons in the fourth instar feeding group with wild plasmid and recombinant plasmid was 12.66% and 5.92%, respectively. The moisture content of cocoons in the fifth instar feeding group with wild plasmid and recombinant plasmid was 6.01% and 6.32%, respectively. Except for the third instar feeding group with recombinant plasmid, which had a lower moisture content than the control group, all other groups had a higher moisture content than the blank control group. The re-humidification rate of the control group was 3.22%. The re-humidification rate of cocoons in the second instar feeding group with wild plasmid was 9.19%, while the second instar feeding groups with 2 μg, 5 μg, and 8 μg oral recombinant plasmid had re-humidification rates of 9.48%, 6.73%, and 8.30%, respectively. The re-humidification rate of cocoons in the third instar feeding group with wild plasmid was 3.24%, while the oral recombinant plasmid drug group had a re-humidification rate of 2.34%. The re-humidification rates of cocoons in the fourth instar feeding group with wild plasmid and recombinant plasmid were 14.49% and 6.29%, respectively. The re-humidification rates of cocoons in the fifth instar feeding group with wild plasmid and recombinant plasmid were 6.39% and 6.75%, respectively. The moisture regains of the blank control group and the third instar feeding groups with both blank and recombinant plasmid were below 3.3%, while the moisture regains of all other groups were above 6.2%. The moisture regains of cocoons in the second, third, and fourth instar feeding groups with 8 μg blank plasmid were higher than those of the same instar groups fed with recombinant plasmid (Figure 7).

### 2.6. Determination of Gel Content

**Figure 8:**
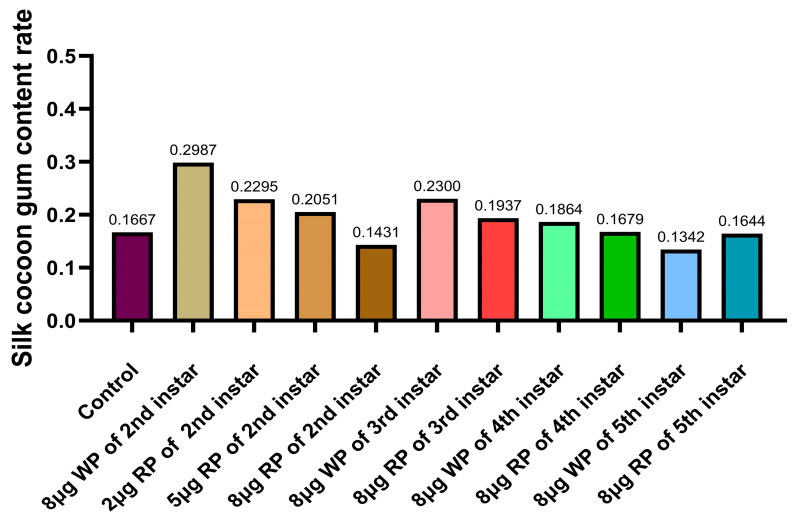
Analysis of the gel content in silkworm cocoons. WP: wild plasmid, RP: recombinant plasmid.

The gel content of the control group was 16.67%. The glue content of cocoons in the second instar feeding group with wild plasmid was 29.87%, the highest among the second instar feeding groups. The gel content of cocoons in the second instar feeding groups with 2 μg, 5 μg, and 8 μg oral recombinant plasmid decreased with increasing dose. The gel content of cocoons in the third instar feeding group with wild plasmid was 23%, while the oral recombinant plasmid drug group had a gel content of 19.37%. The gel content of cocoons in the fourth instar feeding group with wild plasmid and recombinant plasmid was 18.64% and 16.79%, respectively. The gel content of cocoons in the fifth instar feeding group with wild plasmid and recombinant plasmid was lower than that of other groups, at 13.42% and 16.44%, respectively. In the second, third, and fourth instar experiments, the gel content of cocoons in the groups fed with wild plasmid was higher than that in the groups fed with recombinant plasmid (Figure 8).

### 2.7. Measurement of Silk Weight, Length, and Silk Fineness

**Figure 9:**
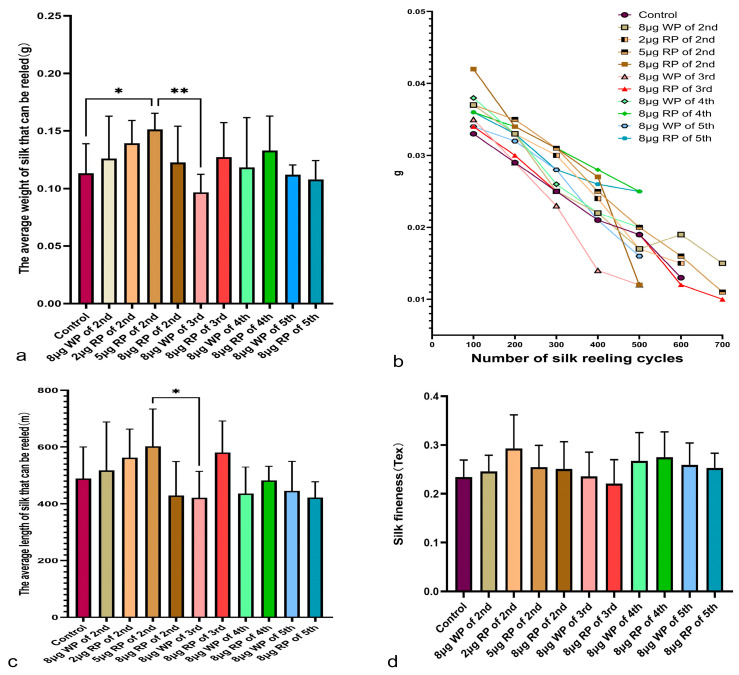
Average weight (**a**), weight of silk that can be reeled every 100 cycles (**b**), average length (**c**), and silk fineness (**d**). Tex = weight (g)/length (m) × 1000. Data are presented as the mean values ± SD, n = 5–15 of groups (valid data of all available cocoons measured). WP: wild plasmid, RP: recombinant plasmid. * *p* < 0.05, ** *p* < 0.01.

In the silk weight measurement data, except for the third instar feeding group with blank plasmid, which had an average silk weight of 0.09672 g, lower than the control group (0.1131 g), all other groups had an average silk weight higher than the control group. The average silk weights of the second to fifth instar feeding groups with recombinant plasmid were higher than those of the same instar groups fed with blank plasmid, except for the second instar feeding group with 8 μg recombinant plasmid. The average silk weight of the second instar feeding group with 5 μg recombinant plasmid was 0.1511 g, the highest among all second instar groups, and there was a significant difference compared to the control group. The average silk weight of the fifth instar feeding group with 8 μg recombinant plasmid was 0.1513 g. In the measurement of silk weight per 100 turns, the control group had the lightest silk weight in the first 100 turns and 200 turns among all groups. The second instar feeding group with 8 μg recombinant plasmid had the heaviest silk weight in the first 100 turns, and heavier silk weights in the 200th, 300th, and 400th turns than most other groups, but the lightest average weight in the 500th turn (Figure 9b).

The trend of average silk length was similar to that of weight, with the average silk lengths of the second to fifth instar feeding groups with recombinant plasmid being longer than those of the same instar groups fed with blank plasmid (503.933 cm), except for the second instar feeding group with 8 μg recombinant plasmid (429.21 cm). The longest average silk length among all the groups was observed in the second instar feeding group with 5 μg recombinant plasmid, at 587.813 cm, followed by the fifth instar feeding group with 8 μg recombinant plasmid, at 583.594 cm (Figure 9c).

### 2.8. SEM Images of the SF Fibers

**Figure 10:**
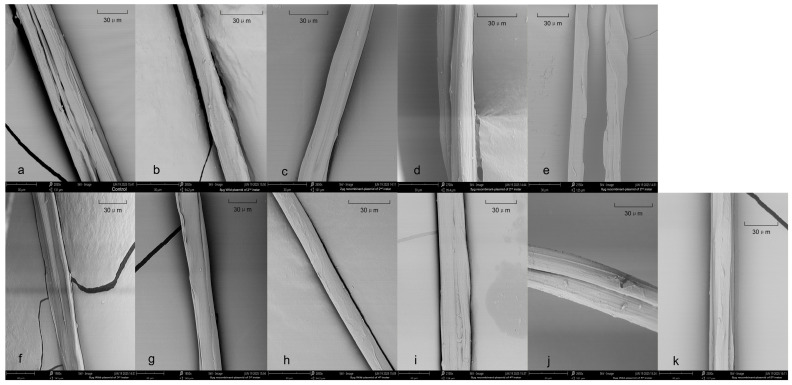
SEM images of silk fibroin fibers from control and plasmid-fed silkworms (bar = 30 µm). (**a**) control group; (**b**) 8 μg wild plasmid of 2nd instar; (**c**) 2 μg recombinant plasmid of 2nd instar; (**d**) 5 μg recombinant plasmid of 2nd instar; (**e**) 8 μg recombinant plasmid of 2nd instar; (**f**) 8 μg wild plasmid of 3rd instar; (**g**) 8 μg recombinant plasmid of 3rd instar; (**h**) 8 μg wild plasmid of 4th instar (**i**) 8 μg recombinant plasmid of 4th instar; (**j**) 8 μg wild plasmid of 5th instar; (**k**) 8 μg recombinant plasmid of 5th instar.

The silk fibers fed with the recombinant plasmid were smoother on the surface than those of the control group and the group fed with a wild-type plasmid. It is speculated that feeding recombinant plasmid improved the fluidity and structural aggregation of silk proteins (Figure 10).

## 3. Discussion

The present study demonstrates that oral administration of a single dose of recombinant pIEx-1-*FKBP12B* plasmid can significantly enhance alkaline phosphatase (ALP) activity and improve key economic traits in the bivoltine silkworm strain Jinqiu × Churi, with the larval instar at the time of feeding being a critical determinant of efficacy. The most profound improvements were consistently observed when the plasmid was delivered during the early larval stages. Overall, the effectiveness of orally administering the pIEx-1-*FKBP12B* plasmid during the larval stages of silkworms followed a distinct pattern: the second instar was superior to the third instar, the third instar was better than the fourth instar, and feeding during the fifth instar yielded poor results. Based on an evaluation of enhanced alkaline phosphatase (ALP) activity, mortality rate, cocooning success rate, cocoon shell weight, cocoon shell ratio, and silk fiber weight and length, administration of the pIEx-1-*FKBP12B* plasmid at the second instar proved most effective.

At present, there have been no reports on oral plasmid delivery in silkworm larvae. Most studies on oral nucleic acid delivery in insects have focused on gene silencing via double-stranded RNA (dsRNA) for pest control. Ghosh et al. soaked leguminous plants in a solution containing genetic material, enabling the invasive pest Halyomorpha halys to take up dsRNA orally by feeding on plant phloem sap, thereby inducing RNA interference [28]. This breakthrough overcame the limitations of traditional injection methods and provided a practical reference for oral transgenic pest control in agriculture. Sáez et al. mixed chitosan-coated plasmid DNA into an artificial diet for beet armyworm (Spodoptera exigua) larvae, confirming that the larvae could successfully absorb the plasmid DNA and synthesize the target protein, with protein yield showing a positive correlation with plasmid DNA concentration [29]. This pioneering work demonstrated the feasibility of oral plasmid delivery in lepidopteran insects. As insects possess potent intestinal nuclease activity [30,31], naked DNA administered orally is generally not viable in the digestive tract. Consistent with this, Sáez et al. failed to recover plasmid-specific sequences or detect reporter gene expression in larvae fed with naked plasmid DNA in an artificial diet [29]. In our study, naked plasmids were uniformly sprayed onto mulberry leaves, and we detected upregulated expression of the target gene *FKBP12B* at the RNA level, as well as the subsequent upregulatory effect of *FKBP12B* on alkaline phosphatase (*ALP*) gene expression and enzyme activity. This phenomenon may be attributed to natural lipid vesicles in mulberry leaves, which encapsulate the plasmids to enhance transfection efficiency and confer partial resistance to the silkworm’s gastrointestinal environment. Supporting this, Ma et al. demonstrated that mulberry leaf-derived lipid vesicles could efficiently deliver GFP and CD98 plasmids while tolerating the human gastrointestinal environment, successfully establishing an oral delivery system [32].

Oral plasmid DNA delivery offers numerous practical advantages for improving cocoon and silk quality, including non-invasiveness, ease of operation, and high safety. Compared to injection, it is non-traumatic, more comfortable for the larvae, and allows for controlled dosing [33]. Sáez et al. confirmed that oral plasmid transfection can persist throughout the continuous developmental stages of larvae, meaning a single administration can achieve sustained recombinant protein expression [29]. Similarly, Turner et al. fed dsRNA to larvae of the lepidopteran Epiphyas postvittana and demonstrated the sustained effect of dsRNA throughout ontogeny [34]. These examples provide strong evidence for the persistence of exogenous nucleic acids in insects across developmental stages following transfection. Our experimental results showed that a single feeding of recombinant plasmids at the second instar stage yielded significantly better outcomes than feeding at the third, fourth, or fifth instar stages. A plausible explanation is that earlier larval stages allow for longer sustained expression of the plasmid-borne gene, resulting in superior efficacy.

Our statistical analysis of alkaline phosphatase activity further showed that an earlier single plasmid administration during the early larval stage resulted in a greater enhancement of this enzymatic activity. This finding aligns with the observed lasting effects of the orally delivered gene [29,34]. In the second instar 5 μg group, the relative expression levels of both s-ALP and m-ALP were significantly increased (3083 and 2894, respectively), and this coordinated upregulation at the RNA level aligned with the measured ALP enzyme activity. This alignment suggests a broad activation of ALP-related physiological pathways, supporting our finding that the second instar 5 μg group achieved the highest ALP activity and the best overall performance. In the third instar group treated with 8 μg of recombinant plasmid, the overall relative ALP expression (s-ALP: 7.252; m-ALP: 0.00866) and ALP activity were higher than in the wild-type plasmid group, reinforcing the link between FKBP12B introduction and ALP activation. Conversely, in the fourth instar group, relative ALP expression at the RNA level was lower than in the wild plasmid group, and ALP activity, while higher than the wild plasmid group, was lower than the control and exhibited a downward trend. An interesting discrepancy was noted in the fifth instar feeding group, where an increase in ALP RNA expression did not translate into higher enzyme activity. This may be because ALP activity in silkworms follows an instar-specific pattern, typically peaking during the high-consumption phase and then dropping sharply before molting [13]—a pattern that appears to dominate in the fifth instar, overriding the plasmid-induced effects seen in younger larvae.

The most common method to enhance ALP activity involves administering hormones, such as 20-hydroxyecdysone (20E) and juvenile hormone (JH). However, hormonal regulation of ALP typically occurs within a short 6–24 h window post-injection, after which the effects dissipate [16]. In contrast, ALP activity in second and third instar larvae fed the recombinant plasmid in our study was still increasing 144 h post-feeding, indicating a sustained effect superior to transient hormone treatment. Furthermore, feeding recombinant plasmid to second-fourth instar larvae also resulted in decreased sericin content and increased silk weight compared to age-matched empty vector groups, whereas feeding in the fifth instar increased sericin content but reduced silk weight.

In their research on Helicoverpa armigera, Zhu Jia et al. speculated that FKBP12 is highly expressed when encountering adverse factors and entering diapause, which in turn inhibits the TOR signaling pathway, enabling Helicoverpa armigera to maintain the diapause state. When diapause is terminated, the expression level of FKBP12 begins to decrease [35]. Knockout of FKBP12 in mammals can upregulate the expression of 4E-BP1. It is known that FKBP12 is an inhibitor of mTOR, and the TOR (target of rapamycin) signaling pathway is a signal transduction pathway that regulates insect body size and nutritional status [36]. The deletion of FKBP12 may increase the activity of mTOR, induce the high expression of 4E-BP1, and enhance cell growth and metabolism [37]. In our research on *Bombyx mori*, the increased expression of FKBP12 can enhance the weight of cocoons and pupae, and even promote the body length growth of fifth instar larvae. Therefore, the regulatory mechanism of FKBP12 in *Bombyx mori* differs from that in Helicoverpa armigera. In the silkworm, FKBP12 protein localizes to both the nucleus and nuclear envelope, whereas in species studied to date, FKBP12 primarily resides in the cytoplasm [19]. Therefore, both the subcellular localization of FKBP12 and its gene function differ from those observed in species studied to date.

This study has several limitations. First, we do not understand the molecular mechanism by which plasmids are orally absorbed in silkworms. Second, the absorption rate of orally administered plasmids in silkworms needs to be improved. Finally, our evidence remains at the transcriptional and enzymatic activity levels; as such, future work should include protein-level validation. Despite these limitations, our findings robustly indicate that oral administration of the pIEx-1-FKBP12B plasmid, particularly during the second instar, is a highly promising strategy for improving silk quality.

## 4. Materials and Methods

### 4.1. Silkworm Rearing and Orally Administering Plasmid

Bivoltine silkworms (Jinqiu × Churi) and fresh mulberry leaves were procured from the “Natural Silkworm Baby Discount Store” on Taobao. Larvae were maintained in an HWS-150B constant temperature and humidity incubator (Changge Mingtu Machinery Equipment Co., Ltd., Xuchang, China) at 26 °C, under a 13-h light/11-h dark cycle and 55% relative humidity. Silkworms were randomly selected and placed in each group. The rearing containers were sterilized plastic culture boxes (10.5 cm × 17 cm × 7.5 cm), with 90 larvae in each box. Fresh leaves were replaced three times a day (08:00, 13:00, and 20:00), and the residual leaves and silkworm droppings were removed in time. When the cocoons were about to form, they were placed in silkworm nets to ensure a comfortable living environment for the animals.

During the 2nd instar, endotoxin-free recombinant plasmid pIEx-1-*FKBP12B* DNA was uniformly sprayed on the mulberry leaves at doses of 2 μg/larva, 5 μg/larva, and 8 μg/larva, and allowed to air dry. The larvae were fed on the second day of the 2nd instar. A control group was fed 8 μg/larva of wild plasmid pIEx-1 DNA, and another completely blank control group was established. Different feeding ages were also tested, with feeding starting on the second day of the 2nd, 3rd, 4th, and 5th instars (8 μg/larva), with 90 silkworm larvae per experimental group (Table 1).

### 4.2. Construction of Overexpressed Recombinant Plasmid

PCR amplification was performed to obtain the target gene (*FKBP12B*) fragment. PCRs were amplified using the following cycling conditions: pre-denaturation at 95 °C for 5 min; 35 cycles of denaturation at 95 °C for 1 min, annealing at 55 °C for 30 s, and extension at 72 °C for 30 s; and a final extension at 72 °C for 10 min [19]. Subsequently, both the amplified product and the plasmid vector were digested with the restriction enzymes *Bam*HI and *Xho*I at the sites incorporated into the primer sequences. The *FKBP12B* gene was subcloned into the pIEx-1 vector. The digested product was recovered, ligated to a plasmid vector (pIEx-1) using ligase, transformed into competent cells (DH5a cells), plated, and cultured overnight. The pIEx-1-*FKBP12B* plasmid was maintained at the College of Life Sciences and Medicine, Zhejiang Sci-Tech University, China. Monoclonal cultivation was performed through preliminary identification of positive clones, PCR, and enzyme digestion for preliminary identification of cloned sequences. The following commercial reagents were used: Taq DNA Polymerase kit and DNase I (Qiagen, Hilden, Germany); PCR Rapid Purification kit (Axygen, Union City, CA, USA); restriction endonucleases (Fermentas, Amherst, NY, USA); T4 DNA ligase and IPTG (Promega, Madison, WI, USA); TRIZOL reagent (Invitrogen, New York, NY, USA); and the Two-step SYBR Green and SYBR^®^ Premix Ex Taq™ kits (TaKaRa, Otsu, Japan). Two primers were designed as follows:

P1: 5′-AAAGGATCCATGGGAGTCGACG-3′ (a BamHI recognition site is underlined);

P2: 5′-GCCCCTCGAGTTATTCAACACGT-3′ (an XhoI recognition site is underlined).

Finally, the pIEx-1-*FKBP12B* plasmid was extracted using the Endo Free Maxi Plasmid Kit (TIANGEN, Beijing, China).

### 4.3. Alkaline Phosphatase (ALP) Activity Determination

Silkworm larvae from each group were randomly sampled at 12 h, 24 h, 48 h, and 6 days after plasmid feeding. Four larvae were sampled each time, accurately weighed, and homogenized in a mortar under liquid nitrogen with physiological saline added at a ratio of 1:9 (*w*/*v*). The homogenate was transferred to a centrifuge tube and centrifuged at 2500 rpm for 10 min. The supernatant was collected for protein concentration determination using a Nano-300 micro-spectrophotometer (ALLSHENG, Hangzhou, China) by measuring the absorbance at 280 nm.

Alkaline phosphatase (ALP) activity was measured using the ALP (AKP) assay kit from Nanjing Jiancheng Bioengineering Institute (Nanjing, China), according to the manufacturer’s instructions. The reaction system is shown in Table 2. After incubation at 37 °C for 15 min, the absorbance at 490 nm was measured using a microplate reader.

ALP activity is expressed in King’s units per gram of tissue protein, where one King’s unit is defined as the amount of enzyme that produces 1 mg of phenol per gram of tissue protein after 15 min of incubation with substrate at 37 °C. The activity was calculated using the following formula:

$$\mathrm{ALP}\;\mathrm{activity}=\frac{A_{\mathrm{sample}}-A_{\mathrm{blank}}}{A_{\mathrm{standard}}-A_{\mathrm{blank}}}\times \frac{C_{\mathrm{standard}}}{C_{\mathrm{pr}}}$$

where $\;C_{\mathrm{standard}}$ = 1 mg/mL and $C_{\mathrm{pr}}$ is the sample protein concentration (g/mL).

### 4.4. RNA Extraction, Reverse Transcription, and Real-Time Fluorescent Quantitative PCR (qRT-PCR)

Total RNA was extracted from intact silkworm bodies randomly sampled from each group (3–4 silkworms per group)and subjected to starvation treatment using the traditional Trizol method, and the quality of RNA was evaluated by measuring the optical density (OD) value. A microvolume spectrophotometer (Nano-300, All Sheng, Hangzhou, China) was used to measure the OD value. Reverse transcription was performed using the Reverse Transcription Kit with dsDNase (Biosharp, Beijing, China), according to the manufacturer’s instructions, with the PCR program set to 37 °C for 30 min and 85 °C for 5 min.

qRT-PCR was performed using the Biosharp Universal SYBR qPCR Master Mix kit (Biosharp, Beijing, China), with *18S rRNA* (GenBank accession no. DQ347470.1) as the internal reference gene. Three replicates per sample.

The relative quantification (RQ) of gene expression was calculated using the 2^−ΔΔCT^ method. The control group used in the formula was the wild plasmid group, which was fed at the same instar as the experimental group. The CT values were collected and analyzed with the ABI Prism 7500 SDS Software v2.0 (Applied Biosystems, Carlsbad, CA, USA).

According to the SYBR Green I real-time PCR method, the CT values were derived using the ABI Prism 7500 SDS Software (Applied Biosystems, USA), and relative quantification was calculated via the following steps:

(1) CT value: The number of cycles before the fluorescence signal in each reaction tube reaches the set threshold;
(2) The value of ΔCT is equal to the CT value of the target gene minus the CT value of the reference gene.

ΔCT = CT_target gene_ − CT_reference genes_ (*18S rRNA* was selected as the reference gene in our experiments) (1)

(3) $\Delta \bar{\mathrm{CT}}$: the average ΔCT value of the negative control group genes;
(4)
(2) $$\Delta \Delta \mathrm{CT}=\Delta \mathrm{CT}-\Delta \bar{\mathrm{CT}}$$
(5) Relative quantification (RQ):
  RQ = 2 ^−ΔΔ^CT

The relative quantification (RQ) of the target gene represents its change in expression in the experimental group relative to the negative control group. An RQ value greater than 1 indicates an upregulation of gene expression, and an RQ value less than 1 indicates a downregulation of gene expression in our animal experiments on silkworms. The primer sequences used in q-PCR are shown in Table 3.

### 4.5. Measurement of Length, Width, Weight, and Cocoon Shell Ratio Statistics

**Figure 11:**
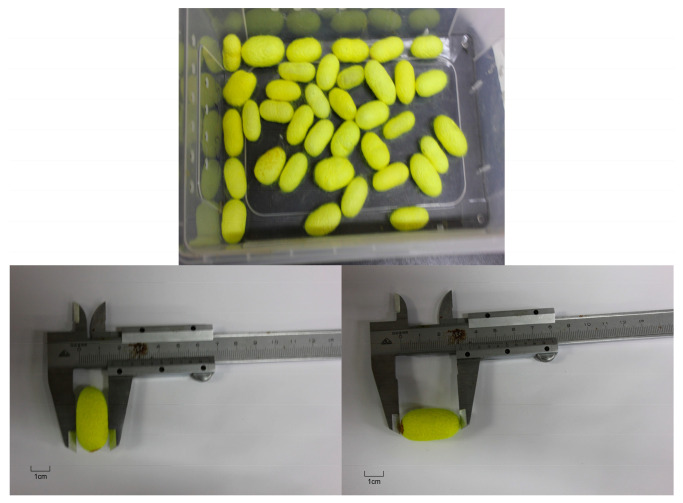
Schematic of silkworm cocoon measurement.

Data were recorded for each 5th instar larva, cocoon, and pupa within each group. The body length of each 5th instar larva was measured on the 5th day, and the length, width, cocoon shell weight, and pupa weight of each cocoon were measured. Cocoon length and width were measured using a vernier caliper (Figure 11). The cocoon shell ratio was calculated by carefully cutting open the cocoon, removing the pupa, weighing the pupa, and subtracting the pupa weight from the total cocoon weight to obtain the cocoon shell weight. The cocoon shell ratio = (cocoon shell weight/total cocoon weight) × 100%. The average cocoon shell ratio, standard deviation, and other statistical parameters were calculated.

### 4.6. Statistical Analysis of Mortality of Larval Stage and Cocoon Formation Rate

The mortality rates were obtained by counting the dead larvae at every instar stage. Then, the mortality of larval and cocoon formation rates of each group was statistically analyzed [38]. The larval mortality rate is the proportion of the number of all dead individuals during the larval stage in a given group to the initial number of larvae in the experiment. The cocoon formation rate is the ratio of successfully cocooned silkworms to surviving late fifth instar larvae in a specific group.

### 4.7. Silk Reeling and Silk Fineness Statistics

The cocoons were collected at 7 days after mounting, boiled in 100 °C water for 3 min, soaked in warm water for 30 s, and repeated until evenly cooked (no white areas). The cooked cocoons were transferred to a 60 °C constant temperature water bath, and a single silk thread was found and manually guided onto a reeling machine (Dongfang Huabo Beijing Technology Co., Ltd., CN61M/CZFY-731, Beijing, China). The machine was slowly wound to reel the silk, with every 100 revolutions (1 sound) counting as 100 turns (112.5 m). After every 100 turns, the silk was dried in a constant temperature and humidity room and weighed, recording the turn count. Silk fineness was measured in Tex units (1 Tex = 1 g of silk per 1000 m), calculated using the following formula: Tex = weight (g)/length (m) × 1000 [39,40].

### 4.8. Re-Humidification Rate and Moisture Content Determination of Silkworm Cocoons

Samples were packaged and conditioned according to GB/T 9995-1997 [41]. The mass of each sample was quickly weighed before drying (accurate to 0.001 g), and the sample was placed in an oven set at 105 °C ± 2 °C until a constant weight was reached (continuous weighing intervals determined using preliminary tests, with weight loss ≥ 98% of the final value). The sample was then weighed after closing the airflow and recorded as the dry weight and the empty container weight.

### 4.9. Determination of Cocoon Gel Content

Ten cocoons were randomly sampled from each group, marked, and dried to a constant weight. The dry weight was recorded before degumming. The cocoons were then placed in a 0.5 g/L sodium carbonate solution and immersed in DI water at 95 °C for 30 min, and then put in cool water for degumming, stirring continuously with a glass rod for uniform degumming. Next, the cocoons were washed with 50–60 °C distilled water three times, dried, and weighed again to obtain the degummed dry weight [4,42,43].

### 4.10. Morphology Characterization

The surface morphologies of the obtained silk fibroin fibers were observed using a scanning electron microscope (JSM-5610LV) (JEOL Ltd., Tokyo, Japan) at 5 kV after sputter-coating with platinum [4,44].

### 4.11. Statistical Analysis

The GraphPad Prism software (version 10) (GraphPad Software, Inc., San Diego, CA, USA) was used for statistical analysis, with data presented as the mean ± standard deviation (SD). Statistical significance was determined using analysis of variance (ANOVA), with *p* < 0.05 considered statistically significant and *p* < 0.01 considered highly significant [39,42]. Significance levels denoted by asterisks follow a hierarchical scale: *p* < 0.001 indicates higher significance than *p* < 0.01, while *p* < 0.0001 indicates higher significance than *p* < 0.001 [39,42].

## 5. Conclusions

In the silkworms fed with a single oral dose of 5 µg recombinant pIEx-1-*FKBP12B* plasmid per larva in the second instar group, overall performance was optimal. The cocoon shell weight was 21.96% higher than the control, the cocoon shell ratio was 2.5% higher than the control, the silk fiber weight was 33.60% higher than the control, and the mortality rate was the lowest among all the groups; in particular, 36.89% lower than the control group. Other performance indicators also showed positive outcomes. These results suggest that orally administering the pIEx-1-*FKBP12B* plasmid has potential application value for improving the quality of silk.

## Figures and Tables

**Table 1:** Sample group setting explanation.

Larval Stages of *Bombyx mori*	Group Names	Quantity
	- control group	150
2nd instar	- wild plasmid pIEx-1 group (8 μg/Larvae) - 2 μg recombinant plasmid pIEx-1-*FKBP12B* group (2 μg/Larvae) - 5 μg recombinant plasmid pIEx-1-*FKBP12B* group (5 μg/Larvae) - 8 μg recombinant plasmid pIEx-1-*FKBP12B* group (8 μg/Larvae)	90
3rd instar	- wild plasmid pIEx-1 (8 μg/Larvae) - 8 μg recombinant plasmid pIEx-1-*FKBP12B* group (8 μg/Larvae)	90
4th instar	- wild plasmid pIEx-1 (8 μg/Larvae) - 8 μg recombinant plasmid pIEx-1-*FKBP12B* group (8 μg/Larvae)	90
5th instar	- wild plasmid pIEx-1 (8 μg/Larvae) - 8 μg recombinant plasmid pIEx-1-*FKBP12B* group (8 μg/Larvae)	90

**Table 2:** Sample addition method.

Reagent	Blank (μL)	Standard (μL)	Sample (μL)
ddH_2_O	10	–	–
0.1 mg/mL phenol std.	–	10	–
Sample	–	–	10
Buffer	50	50	50
Substrate	50	50	50
*Incubate 37 °C, 15 min*			
Color developer	150	150	150

**Table 3:** Primer sequences in q-PCR.

Gene	Sequence of Forward Primer (5′-3′)	Sequence of Reverse Primer (5′-3′)
*FKBP12B*	TTCACCTGGAAATGGACAACTTA	TTTCCCCTACAGACATCTTTGCTA
*s-ALP*	GAACGATGGGCGAAATCTGA	GGGTTGGCTCCGTGGTATG
*m-ALP*	CGTAACGACCGACGCCAACT	CATTGATGCCGCAAGGTGA
*18s rRNA*	CGATCCGCCGACGTTACTACA	GTCCGGGCCTGGTGAGATTT

## Data Availability

The original contributions presented in this study are included in the article. Further inquiries can be directed to the corresponding author.
